# Microscopic features of small bowel mucosa of patients with Crohn’s disease

**DOI:** 10.1186/s12876-019-1138-2

**Published:** 2019-12-30

**Authors:** Yun Cui, Shi-yuan Lu, Jie Xu, Yan-shen Peng, Qi Miao, Xiao-qing Wang, Xiao-yu Chen, Zhi-hua Ran

**Affiliations:** State Key Laboratory for Oncogenes and Related Genes, Key Laboratory of Gastroenterology and Hepatology, Ministry of Health, Division of Gastroenterology and Hepatology, Shanghai Institute of Digestive Disease, Renji Hospital, School of Medicine, Shanghai Jiao Tong University, 145 Middle Shandong Road, Shanghai, 200001 China

**Keywords:** Crohn’s disease, Granulomatous lymphangitis, Histopathology

## Abstract

**Background:**

Double-balloon enteroscopy enables performing numerous small bowel biopsies for pathologic analysis. However, most histopathological characteristics of Crohn’s disease are non-specific characteristics. We aimed to explore the small bowel mucosal histopathologic characters of Crohn’s disease and identify some disease-specific changes.

**Methods:**

We included 253 patients without tumors and grouped them into Crohn’s disease, suspected Crohn’s disease, and non-Crohn’s disease groups. These patients underwent double-balloon endoscopy examination and small bowel biopsy at Renji Hospital, Shanghai. All histopathological sections were reviewed, and > 20 histopathological parameters were assessed. Immunohistochemistry was conducted when necessary.

**Results:**

There were different forms of granulomatous lymphangitis on the small bowel mucosa in Crohn’s disease. They showed as various macrophages or epithelioid cells in the lumina of lymphatics or in the center of the villi with or without evident obstruction. These features were only observed in Crohn’s disease patients. Furthermore, they were correlated with granuloma and lymphangiectasia. Additionally, 15 other features showed significant differences among the three groups, and Crohn’s disease patients showed an average of almost seven histopathological characteristics.

**Conclusions:**

We described the detailed morphologies of granulomatous lymphangitis on the small bowel mucosa and recommend it as a useful histopathological feature for the diagnosis of Crohn’s disease. In terms of specificity and sensitivity, it was superior to non-caseating epithelioid granuloma.

## Background

Crohn’s disease (CD) is a chronic, relapsing, inflammatory intestinal disorder and affects numerous individuals worldwide. Currently, its occurrence is considered stable or decreasing in Western countries; however, its incidence has been dramatically rising in China [1–3] .

Although small bowel is the most commonly affected site, CD can affect any part of the digestive tract. Double-balloon enteroscopy is useful in exploring small bowel lesions [4, 5]. The increasing use of double-balloon enteroscopy has enabled performing multiple small bowel biopsies for microscopic analysis. Non-caseating epithelioid granuloma is a key microscopic feature for the diagnosis and differential diagnosis of CD [6, 7]. It is formed by compact aggregation of epithelioid histocytes without central necrosis. Generally, it is smaller than 400 μm and non-confluent. Other features indicative of CD include transmural inflammation, multiple lymphoid aggregations, fissuring ulcers, submucosal fibrosis, and neural hyperplasia [8]. However, although most of these changes were easily identifiable on surgical specimens, they usually did not present on biopsy tissues. Alternatively, some other features identified on the small bowel mucosa were reported, such as chronic inflammation, aphthous ulcers, architectural aberrant, pyloric gland metaplasia, surface irregularities, and basal plasmacytosis [9–13].

Environmental factors, genetic predisposition, dysregulated mucosal immune responses, and intestinal flora are reportedly involved in the pathogenesis of CD [14–16]. Further, pathologists have suggested lymphocytic and granulomatous lymphangitis as the fundamental alteration of CD [17]. A dense network of lymphocytes, histocytes, and macrophages within the lymphatic system results in the obstruction of regional lymphatics. This complex structure was observed in all layers of the intestinal wall in CD [18]. Coincidently, transmural inflammation, multiple lymphoid aggregations in the submucosa, and beaded changes of the serosa occurred only where the lymphatics were located. This suggests granulomatous lymphangitis as the underlying physiopathological mechanism of CD. Consequently, it could be a potential histopathological feature for CD. However, it has not attracted much attention from other pathologists thus far, and its detailed morphology remains to be fully illustrated.

Although many pathologic changes, including granuloma, could be seen on the small bowel mucosa of CD, all of them were non-specific. Herein, we reviewed the mucosal histopathological features of CD and tried to identify some essential, CD-specific changes.

## Methods

This study included 305 consecutive patients who underwent double-balloon endoscopy and small bowel biopsy at Renji Hospital of the Shanghai Jiaotong University between January 2016 and August 2017. Among these patients, we excluded those whose small bowel biopsy tissues showed neoplastic lesions, including intraepithelial neoplasia (seven cases), carcinoma (three cases), lymphoma or lymphoid proliferative disease (14 cases). Moreover, we also excluded cases showing only necrosis, inflammatory exudates, or granulation tissues without a small bowel mucosa component on biopsy samples (18 cases). Ten patients whose biopsy specimens were too small for evaluation were also excluded. Finally, 253 patients (men, 186) without tumors were enrolled in our study. The overall mean age was 35.96 years (14–75 years). Based on the final clinical diagnosis, we divided the patients into three groups: CD group, suspected CD group, and non-CD group. CD was diagnosed based on the diagnostic criteria mentioned in the 3rd European evidence-based consensus on the diagnosis and management of Crohn’s disease (2016) [19].

All histopathological sections were retrieved and reviewed by the same experienced pathologist. Tissue blocks were selected for necessary immunohistochemistry analyses. The pathologist, while evaluating the histopathological features, was blinded to the patient’s clinical and grouping information. The following histopathological parameters (22 in total) were analyzed: the inflammation depth and distribution, cryptitis/crypt abscesses, intra-superficial epithelium neutrophils, neutrophils in the lamina propria, basal plasmacytosis, erosion, pyloric gland metaplasia, crypt architectural abnormality (including distortion, non-parallel structure, irregularity, diameter change, dilation, branching, extension, and reduction in number), villous architecture abnormality (including shortening, widening, flattening, and blunting), increased goblet cell count, decreased intraepithelial mucus, lymphangiectasia, thickened muscularis mucosa, lymphoid aggregation in the mucosa and submucosa, fibrous tissue in the submucosa, ganglion cells, granulation tissues, granuloma (excluding those related to crypt injury), and granulomatous lymphangitis. In addition, we attempted to evaluate other identifiable structures. D2–40 (Shanghai Long Island Biotec, China) immunohistochemistry staining was as per the manufacturer’s protocol to outline the shapes of lymphatics, and CD68 antibody (Shanghai Long Island Biotec, China) was used to show histocytes and macrophages.

Data were shown as mean ± SD. Student’s t-test and non-parametric test (Kruskal–Wallis H test), chi-square test, and Spearman’s correlation were performed using SPSS19.0 software.

## Results

### General clinical information

Table 1 shows the spectrum of diseases in the non-CD group and patients’ general clinical information. The general clinical information of CD patients such as the main clinical presentation, enteroscopic and radiological changes were summarized in Additional file 1: Table S1. The comparison of patients’ clinical information among the three groups is shown in Table 2. The mean age of CD patients was approximately 6 years lesser than that of non-CD patients. There were no significant differences in the biopsy site (ileum and jejunum) and biopsy depth among the three groups.

**Table 1:** The clinical information of the non-CD patients

Clinical diagnosis	Total cases(ratio)	Male cases	Female cases	Age(y) Mean ± SD
Suspected vasculitis	5(5.21%)	2	3	23.40 ± 5.94
Small bowel ulcer	36(37.50%)	29	7	44.22 ± 16.06
Small bowel inflammation	32(33.33%)	23	9	34.88 ± 16.58
Digestive tract bleeding	7(7.29%)	6	1	31.00 ± 7.78
Digestive tract occupation lesions	5(5.21%)	1	4	51.00 ± 10.70
Diverticulum	1(1.04%)	1	0	60.00
Irritable bowel syndrome	4(4.17%)	4	0	36.25 ± 19.08
Polypoid changes	5(5.21%)	4	1	41.60 ± 17.60
Vascular malformation	1(1.04%)	0	1	42.00

**Table 2:** The patients’ clinical information among the three groups

	CD cases(ratio)	suspected CD cases(ratio)	non-CD cases(ratio)	P
Cases	137	20	96	
Sex				0.902
male	102(74.50%)	14(70.00%)	70(72.92%)	
female	35(25.50%)	6(30.00%)	26(27.08%)	
Age(y)	15–69	19–59	14–75	0.020
(Mean ± SD)	(33.74 ± 12.82)	(36.10 ± 11.83)	(39.08 ± 16.31)	
Method^†^				0.020
via mouth	40(29.20%)	11(55.00%)	41(42.71%)	
via anus	97(70.80%)	9(45.00%)	55(57.29%)	
Biopsy site				
ileum	108(78.83%)	11(55.00%)	67(69.79%)	0.004^a^
jejunum	37(27.01%)	10(50.00%)	34(35.42%)	
duodenum	0(0.00%)	0(0.00%)	7(7.29%)	
Biopsy depth				0.186
M ^‡^	28(20.44%)	6(30.00%)	32(33.33%)	
MM ^‡^	67(48.90%)	10(50.00%)	36(37.50%)	
SM ^‡^	42(30.66%)	4(20.00%)	28(29.17%)	

†:Double balloon enteroscopy examination method.

‡:M:mucosa;SM:submucosa;MM:muscularis mucosa.

^a^There were no significant differences between ileum and jejunum biopsy sites

### Microscopic characteristics

**Fig. 1:**
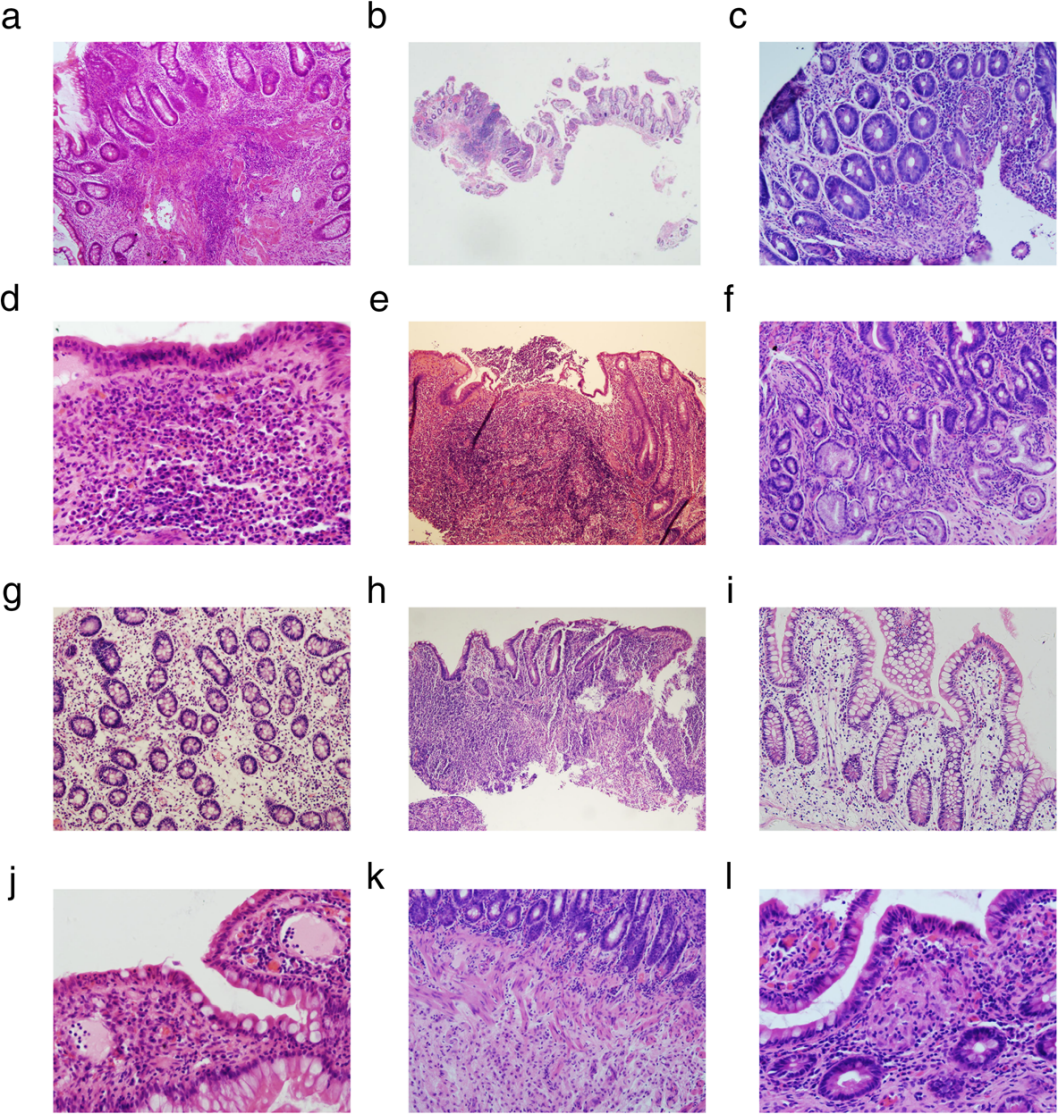
Fifteen microscopic features that showed significant differences among the three groups. **a**: Inflammation involved the submucosa and basal plasmacytosis. HE 100×; **b**: Uneven inflammation. Inflammation on the left was severe and the right was mild. HE 20×; **c**: Cryptitis (lower left corner) and crypt abscesses (upper right corner). HE 200×; **d**: Intra-surface epithelium neutrophil and intra-mucosa neutrophil. HE 400×; **e**: Erosion showing as superficial epithelial damage. HE 100×; **f**: Pyloric gland metaplasia with pale cytoplasm. HE 200×; **g**: Aberrant crypt structure: distorted, non-parallel, and irregular. HE 200×; **h**: Villi changes (shortening, widening, and blunting) and decrease in intra-epithelial mucus. HE 100×; **I**: Increased goblet cells. HE 200×; **J**: Lymphangiectasia. HE 400×; **k**: Thickened muscularis mucosa. HE 200×; **l**: non-caseating epithelioid granuloma. HE 400×

The rates of microscopic characteristics among the three groups are shown in Table 3. Among the 21 microscopic changes (listed in Table 3), 15 features significantly differed among the three groups. Histopathological morphologies of these 15 features are shown in Fig. 1.

**Table 3:** The microscopic features of the small bowel mucosa among the three groups

		CD cases(ratio)	suspected CD cases(ratio)	non-CD cases(ratio)	P
Cases		137	20	96	
Histopathological parameters					
No.	Microscopic features				
1	Inflammation depth				0.001
	SM^†^	10(7.30%)	0(0.00%)	3(3.13%)	
	M^†^	127(92.70%)	20(100.00%)	93(96.87%)	
2	Uneven inflammation	114(83.21%)	10(50.00%)	38(39.58%)	0.000
3	Cryptisis/Crypt abscesses	42(30.66%)	4(20.00%)	15(15.63%)	0.029
4	Intraepithelium neutrophil	41(29.93%)	4(20.00%)	10(10.42%)	0.002
5	Intramucosa neutrophil	51(37.23%)	6(30.00%)	17(17.71%)	0.006
6	Capillaries increase	14(10.22%)	2(10.00%)	7(7.29%)	0.797
7	Basal plasmacytosis	25(18.25%)	1(5.00%)	8(8.33%)	0.041
8	Erosion	39(28.47%)	3(15.00%)	14(14.58%)	0.029
9	Pyloric gland metaplasia	30(21.90%)	7(35.00%)	7(7.29%)	0.001
10	Abberant crypt stucture	120(87.59%)	16(80.00%)	52(54.17%)	0.000
11	Villus changes	114(83.21%)	14(70.00%)	50(52.08%)	0.000
12	Goblet cells increase	83(60.58%)	9(45.00%)	17(17.71%)	0.000
13	Intraepithelium mucus decrease	34(24.82%)	7(35.00%)	11(11.46%)	0.011
14	Lymphangiectasis	47(34.30%)	4(20.00%)	10(10.42%)	0.000
15	Thickened muscularis mucosa	54(39.42%)	2(10.00%)	15(15.63%)	0.000
16	SM-LC aggregation^†^	15(35.70%)	2(50.00%)	9(32.10%)	0.720^a^
17	M-LC aggregation^†^	25(18.25%)	2(10.00%)	24(25.00%)	0.253
18	SM fibrosis^†^	6(14.30%)	0(0.00%)	2(7.10%)	0.480^a^
19	Ganglion cells	18(13.14%)	1(5.00%)	5(5.21%)	0.100
20	Granulation tissue	14(10.22%)	2(10.00%)	5(5.21%)	0.425
21	Granuloma	24(17.52%)	3(15.00%)	2(2.08%)	0.001
	Granulomatous lymphangitis	33(24.09%)	1(5.00%)	0(0.00%)	0.078^b^
Number of parameters		6.96 ± 3.73	5.1 ± 3.86	3.46 ± 3.39	0.000

†:*M* mucosa;*SM* submucosa;*LC* lymphocyte.

^a^The cases pool were those of which biopsy depth was deep enough to submucosa

^b^There was no significant difference between CD and suspected CD groups

**Fig. 2:**
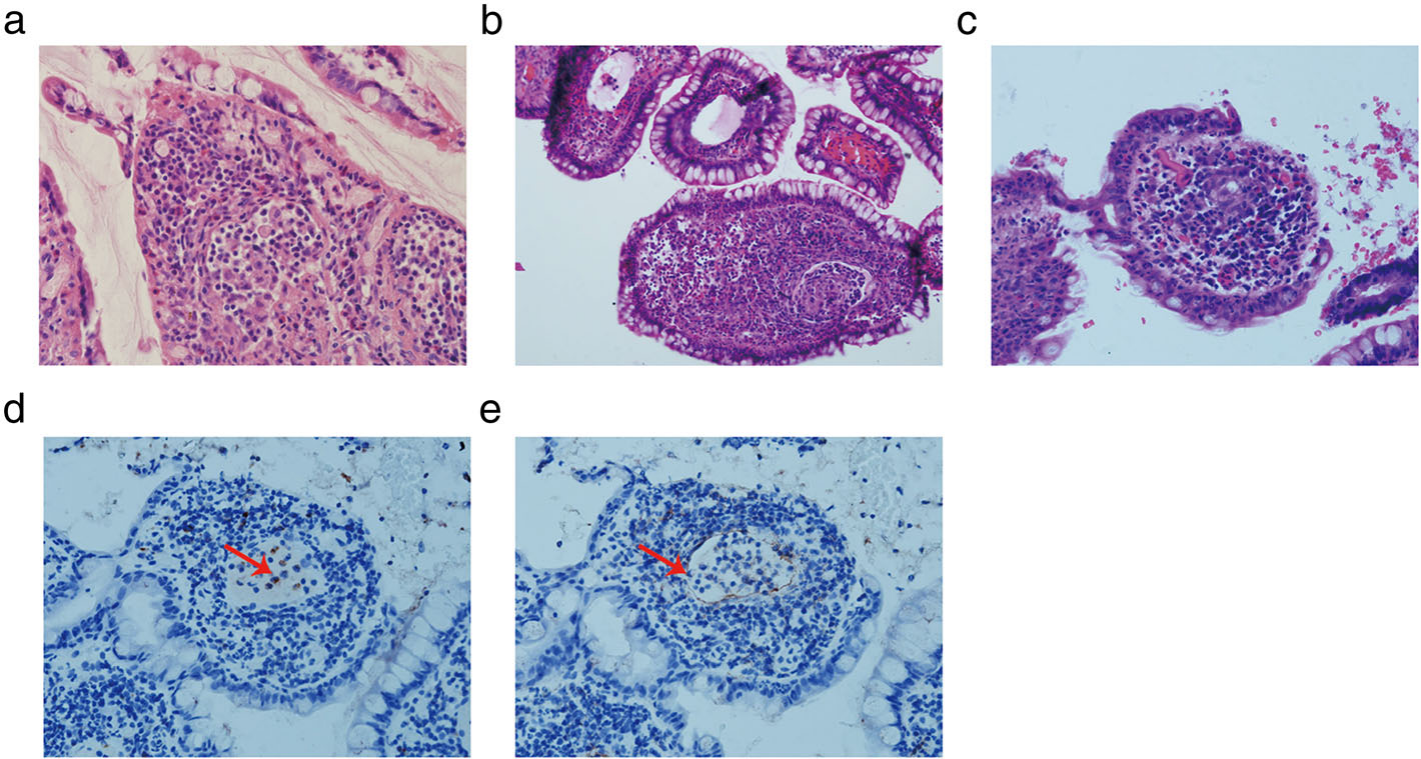
Obstructive granulomatous lymphangitis. **a**: Obstructive granulomatous lymphangitis with scattered macrophages in the lymphatics. HE 400×; **b**: Obstructive granulomatous lymphangitis with compacted macrophages in the lymphatics. HE 400×; **c**: A few epithelioid cells aggregated in the center of the villus. HE 400×; **d**: CD68 immunohistochemistry showing macrophages scattered in the lumina of the lymphatics (as shown by the red arrow). 400×; **e**: D2–40 immunohistochemistry outlined the very thin lymphatic vessels (as shown by the red arrow). 400×

**Fig. 3:**
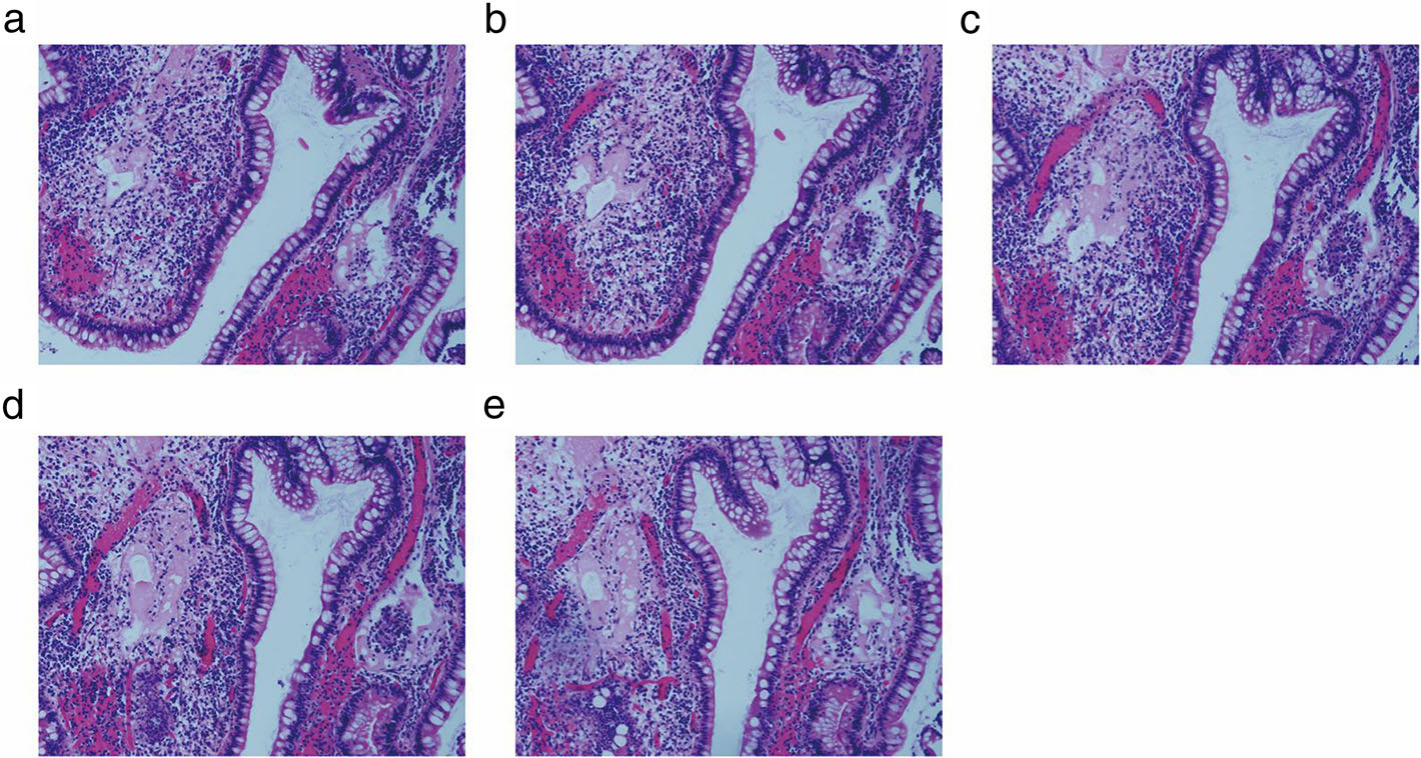
Granulomatous lymphangitis without complete obstruction. **a**: A few lymphocytes and macrophages aggregated in the lumina of the lymphatics in the villus. HE 200×; **b**, **c**, **d**, and **e** were serial sections of the same tissue as A with 4 μm per level. HE 400×

We noticed that granulomatous lymphangitis had diverse morphologies on the small bowel mucosa. The classic form was wherein macrophages or epithelioid cells filled in and obstructed the lymphatics. Lymphocytes usually appeared around or mixed with these cells. The narrow space around the epithelioid cells manifested that they were within the lymphatics. Scattered and/or compacted macrophages completely blocked the lymphatics. The scattered macrophages had round or oval nucleus with ample pale or pink cytoplasm around, and some of these cells tended to adhere to each other, showing a pattern that is otherwise characteristic of epithelial cells (Fig. 2a). The compacted macrophages were with eosinophilic cytoplasm and oval or rod-shaped nucleus just like those in non-caseating epithelioid granuloma (Fig. 2b). On transverse sections, particularly those atop the villi, the epithelioid cell mass became very small and the lymphatics or the lacteal wall could not be easily found (Fig. 2c). However, on deeper sections, it was seen that this cells mass connected to a dilated lymphatic within which there were several macrophages and lymphocytes similarly as typically seen in granulomatous lymphangitis. Macrophages with CD68 expression were in the lumina, whereas D2–40 immunohistochemistry clearly outlined the shape of the lymphatic vessels and the lacteal of villi (Figs. 2d and e). The second form of granulomatous lymphangitis presented as a mass of cells just floating in the lumina of lymphatics with incomplete obstruction (Figs. 3a-e). Serial sections manifested the volume changing of the cell mass in the lymphatics. Unlike in the first form, in the second form, even the biggest cell mass did not completely block the lymphatics. These cells usually showed eosinophilic cytoplasm. In our study, almost all cases of granulomatous lymphangitis showed the location to be the lacteals of villi. We identified that one suspected CD patient and 33 CD patients presented with granulomatous lymphangitis, whereas none of the non-CD patients showed this presentation. In the CD group, 33/137 (24.09%) patients presented with granulomatous lymphangitis.

Table 4 shows a comparison of the granuloma’s characters among the three groups. All granulomas were non-caseating epithelioid granulomas. The mean granuloma size in the CD group was 154.57 ± 60.80 μm, with half of them being larger than 200 μm (12 cases) and the other half being smaller than 200 μm (12 cases). The largest granuloma in the CD group measured 305 μm. However, there were no significant differences in granuloma distribution, site, size, and the number of granulomas per case among the three groups.

**Table 4:** Granuloma characters among the three groups

	CD cases(ratio)	suspected CD cases(ratio)	non-CD cases(ratio)	P
Cases^†^	24	3	2	
Granuloma distribution				0.761
villus	14(58.33%)	3(100.00%)	1(50.00%)	
area besides villi	7(29.17%)	0(0.00%)	1(50.00%)	
SM^‡^	3(12.50%)	0(0.00%)	0(0.00%)	
Granuloma site				0.446
ileum	22(91.67%)	2(66.67%)	2(100.00%)	
jejunum	2(8.33%)	1(33.33%)	0(0.00%)	
Mean size±SD (um)	154.57 ± 60.80	96.67 ± 18.15	90 ± 15.23	0.098
Numbers/cases	1.46 ± 1.14	1.333 ± 0.58	2.5 ± 2.12	0.467

†:The cases pool were those which had granulomas on biopsy tissues.

‡:submucosa.

Further, two correlations of granulomatous lymphangitis were identified, one with granuloma and the other with lymphangiectasia. From Tables 5 and 6, we can see that more cases with granulomatous lymphangitis showed granulomas (23.53%, 8/34) and more cases with lymphangiectasia were shown as having granulomatous lymphangitis (34.43%, 21/61) in the mucosa.

**Table 5:** The correlation between granulomatous lymphangitis and granuloma

		Granuloma				Spearman’s correlation	
		+	–	*n*	*P*	r	*P*
Granulomatous lymphangitis	+	8	26	253	0.018	0.149	0.018
	–	21	198

**Table 6:** The correlation between granulomatous lymphangitis and lymphangiectasia

		Lymphangiectasia				Spearman’s correlation	
		+	–	*n*	*P*	r	*P*
Granulomatous lymphangitis	+	21	13	253	0.000	0.347	0.000
	–	40	179				

## Discussion

We observed that granulomatous lymphangitis showed as different forms in the small bowel mucosa of CD. One form was the classic obstructive granulomatous lymphangitis with scattered or compacted macrophages filled in the lymphatics. This presentation was similar to that described in previous researches [17, 18, 20]. Herein, we also presented one change atop the villi wherein macrophages or epithelioid cells aggregated in the center of the villi with no clear lymphatics around. However, these cells were confirmed by immunohistochemistry to connect to a lymphatic vessel on deeper sections with granulomatous lymphangitis. Therefore, this change could be a type of granulomatous lymphangitis. The lymphatic endothelial cells around may be too tender for visualization, too damaged, or mixed with surrounding crowed cells, as previously reported [18]. The other form of granulomatous lymphangitis showed no evident obstruction. Macrophages or epithelioid cells were not found to always obstruct the lymphatics completely. Sometimes, these cells were present in the lumina merely as a sign of granulomatous lymphangitis. The non-obstructive manifestation may be an earlier stage. We considered that all these forms of granulomatous lymphangitis in our study to be significant as all but one patient with these structures belonged to the CD group. The one exception in the suspected CD group was already confirmed as having CD on follow-up examination in February 2019. Therefore, granulomatous lymphangitis on small bowel mucosa may be a feature specific to CD, but more prospective studies in large cohort are needed. Our study reinforced the theoretical findings reported in Dr. Van Kruiningen’s research [17] and also described the detailed morphologies of granulomatous lymphangitis. In our study, it was helpful for the pathologist to consider the histopathological morphologies of granulomatous lymphangitis on hematoxylin and eosin-stained sections in the diagnosis and differential diagnosis of CD. Immunohistochemistry was usually not necessary as the small number of macrophages or epithelioid cells and the lymphatic vessels were generally too tender to be captured on deeper level sections, particularly for the small bowel biopsies.

Granulomatous lymphangitis was identified in the mucosa in 24.09% of CD patients in our study. This rate of occurrence was higher than that previously reported by Sura [20]. This can be explained by the granulomatous lymphangitis presenting as multiple forms in the mucosa. It may also be because the threshold of quantities of macrophages or epithelioid cells in dilated lymphatics was different. In the diagnosis and differential diagnosis of CD, non-caseating epithelioid granuloma was typically considered an important microscopic feature. In our study, 17.52% of CD patients had non-caseating epithelioid granuloma on biopsy tissues, and this occurrence rate was significantly greater than that in other groups. However, regarding specificity and sensitivity, our data suggested that granulomatous lymphangitis was a better microscopic feature than non-caseating epithelioid granuloma for CD.

Furthermore, we showed that granulomatous lymphangitis was significantly correlated with both granuloma and lymphangiectasia. A higher occurrence rate of granuloma was found in patients with granulomatous lymphangitis, and a higher rate of granulomatous lymphangitis was found in patients with lymphangiectasia. Therefore, lymphangiectasia could be a useful indicator of granuloma and granulomatous lymphangitis. We recommended pathologists to put more efforts and physicians to take more biopsies to enhance the chances of finding granulomas and granulomatous lymphangitis when encountering cases with lymphangiectasia in the small bowel mucosa.

Although other microscopic features like aberrant crypt structure, uneven inflammation, and villi changes were common in CD patients, they were widely present in the other two groups as well, suggesting that these microscopic features are quite non-specific and not reliable when making a diagnosis of CD. Herein, CD patients showed an average of almost seven histopathological characteristics in the small bowel mucosa. Suspected CD and non-CD patients showed significantly fewer characteristics. When small bowel biopsy revealed the absence of granulomatous lymphangitis and granuloma, more than six histopathological characteristics may be histopathologically considered as points of reference.

In addition, our study presented the proportion of diseases among patients who underwent double-balloon enteroscopy during a certain period. The proportion of diseases in our study has an evident local characteristic that more than half cases were with CD. Intestinal tuberculosis (ITB) was an important condition that occasionally confused pathologists and physicians and obfuscated the diagnosis of CD. Though few studies have reported on the presence of granulomatous lymphangitis in human ITB, it was necessary for it to be evaluated in both ITB and other granulomatous diseases before confirming its diagnostic value for CD.

## Conclusions

We described in detail the morphologies of granulomatous lymphangitis in the small bowel mucosa and are, to our knowledge, the first to highlight its roles in the diagnosis of CD. In terms of specificity and sensitivity, granulomatous lymphangitis was superior to non-caseating epithelioid granuloma. We expect these findings to have significant impact on daily clinicopathological work.

## Supplementary information

**Additional file 1: Table S1.** The clinical information of CD patients

## Data Availability

The datasets used and analyzed during the current study available from the corresponding author on reasonable request.

## Abbreviation

CD: Crohn’s disease
